# Adherence to the Mediterranean diet to prevent or delay hepatic steatosis: a longitudinal analysis within the PREDIMED study

**DOI:** 10.3389/fnut.2025.1518082

**Published:** 2025-05-21

**Authors:** Raquel Cueto-Galán, Andres Fontalba-Navas, Mario Gutiérrez-Bedmar, Miguel Ruiz-Canela, Miguel A. Martínez-González, Lilian Alves, Nancy Babio, Montserrat Fitó, Emilio Ros, Miquel Fiol, Ramón Estruch, Fernando Arós, Luis Serra-Majem, Xavier Pintó, Carlos Muñoz-Bravo, Antonio García-Rodríguez, Enrique Gómez-Gracia

**Affiliations:** ^1^Department of Public Health and Psychiatry, School of Medicine, University of Málaga, Málaga, Spain; ^2^Biomedical Research Institute of Malaga (IBIMA), Málaga, Spain; ^3^Antequera Hospital, Northern Málaga Integrated Healthcare Area, Antequera, Spain; ^4^CIBER Cardiovascular Diseases (CIBERCV), Instituto de Salud Carlos III (ISCIII), Madrid, Spain; ^5^Department of Preventive Medicine and Public Health, School of Medicine, University of Navarra, Pamplona, Spain; ^6^CIBER Fisiopatología de la Obesidad y Nutrición (CIBEROBN), Instituto de Salud Carlos III (ISCIII), Madrid, Spain; ^7^Department of Nutrition, Harvard T.H. Chan School of Public Health, Boston, MA, United States; ^8^Gastroenterology Department, Hospital Salut Sant Joan, Institut d'Investigació Sanitària Pere Virgili, Food, Nutrition, Development, and Mental Health Group, Reus, Spain; ^9^Universitat Rovira i Virgili, Departament de Bioquímica i Biotecnologia, Grupo ANUT-DSM, Reus, Spain; ^10^Institut d'Investigació Sanitària Pere Virgili, Reus, Spain; ^11^Cardiovascular Risk and Nutrition Research Group, Hospital Del Mar Medical Research Institute (IMIM), Barcelona, Spain; ^12^Lipid Clinic, Endocrinology and Nutrition Service, Institut d'Investigations Biomèdiques August Pi I Sunyer (IDIBAPS), Hospital Clínic, Barcelona, Spain; ^13^Health Research Institute of the Balearic Islands (IdISBa), Hospital Son Espases, Palma de Mallorca, Spain; ^14^Department of Internal Medicine, IDIBAPS, Hospital Clínic, University of Barcelona, Barcelona, Spain; ^15^Department of Cardiology, Hospital Universitario de Álava, Vitoria, Spain; ^16^Department of Clinical Sciences and Research Institute of Biomedical and Health Sciences, Universidad de Las Palmas de Gran Canaria, Las Palmas, Spain; ^17^Lipid and Vascular Risk Unit, Internal Medicine Service, Hospital Universitario de Bellvitge, L'Hospitalet de Llobregat, Spain

**Keywords:** PREDIMED, dietary adherence, Mediterranean diet, randomized controlled trial, metabolic dysfunction-associated steatotic liver disease, hepatic steatosis index

## Abstract

**Background:**

Little is known about the potential preventive effect of adherence to the Mediterranean diet (MedDiet) on the development of metabolic dysfunction-associated steatotic liver disease (MASLD).

**Aim:**

This study aims to determine the impact of adherence to the MedDiet on the progression of MASLD, measured using the hepatic steatosis index (HSI) at baseline and annually over a 5-year follow-up period within the framework of the *PREvención con DIeta MEDiterránea* (PREDIMED) study.

**Method:**

Participants from the PREDIMED trial with sufficient available data (*n* = 3,145) were examined annually over 5 years. Adherence to the MedDiet was evaluated using the Mediterranean Diet Adherence Screener (MEDAS) questionnaire, and the presence/severity of hepatic steatosis was determined according to the HSI. Linear mixed models were used to analyze the association between the study variables and HSI.

**Results:**

The participants (57% female, 43% male) had a mean age of 67.2 (SD 6.2) years. Among the cardiovascular risk factors considered, the mean BMI was 29.81 (SD 3.62); 47% of participants had type 2 diabetes, 70% had hypercholesterolaemia, and 84% had hypertension. Over the 5-year follow-up, average adherence to the MedDiet and physical activity generally increased, while alcohol consumption, calorie intake, tobacco use, hypercholesterolaemia, and hypertension decreased. The fully adjusted multivariate model reflected a statistically significant decrease in the HSI per unit increase in adherence to the MedDiet (β = −0.075; 95% CI: −0.128, −0.021).

**Conclusion:**

In individuals at high cardiovascular risk, adherence to the MedDiet is significantly associated with improvements in HSI. These longitudinal findings highlight the important role of the MedDiet in delaying or slowing the natural progression of MASLD, contributing to both its prevention and clinical management.

## Introduction

The nomenclature for the condition formerly known as non-alcoholic fatty liver disease (NAFLD) has evolved significantly. Traditionally, NAFLD was defined as a fatty accumulation in the liver (steatosis ≥5% of hepatocytes) in the absence of significant alcohol consumption (<20–30 g/day of alcohol intake), liver viral infection, or relevant medication/drug use (1). In 2020, Eslam et al. (2) introduced the term metabolic dysfunction-associated fatty liver disease (MAFLD), characterized by hepatic steatosis identified through imaging, blood biomarkers, or liver histology. Diagnosis requires the individual to be overweight or obese, to have type 2 diabetes mellitus (T2D), or to exhibit at least two metabolic risk factors. In 2023, an internationally guided Delphi process was conducted to standardize diagnostic criteria and enhance the comparability of research findings. This process led to the definition of metabolic dysfunction-associated steatotic liver disease (MASLD) (3), in which the term “steatotic” rather than “fatty” was used to mitigate the stigma associated with the condition (4). Up to 99% of individuals diagnosed with NAFLD also meet the criteria for MASLD (5).

MASLD is defined as steatotic liver disease occurring in the presence of one or more cardiometabolic risk factors and with alcohol intake < 20 g/day (6). However, MASLD may coexist with moderate alcohol intake, resulting in a distinct condition known as metabolic dysfunction-associated alcohol-related liver disease (MetALD).

MetALD represents a separate category from “pure” MASLD, where alcohol consumption plays a significant role in the development and progression of the disease. It is characterized by a weekly alcohol intake of 140–350 g for women and 210–420 g for men, exceeding the threshold for MASLD but not meeting the criteria for alcoholic liver disease (7).

MASLD is the most common liver disease, with a global prevalence of 38.7% (8, 9), ranging from 55.33% in Europe to 36.31% in Asia and 35.99% in North America (10). The condition is pandemic, transcending national and regional differences in economic development and socioeconomic status. Moreover, it is a major risk factor for hepatocellular carcinoma and the third overall cause of death from cancer (8.3%) (11). The accumulation of free fatty acids and triglycerides in the liver generates oxidative stress and inflammation, facilitating the progression to steatohepatitis, fibrosis, and liver cancer (12, 13). Moreover, the rising prevalence of obesity, T2D, and metabolic syndrome has significantly contributed to the growing burden of MASLD (14). The coexistence of these conditions further amplifies the risk and severity of MASLD, making it an increasingly important risk factor for cardiovascular events (15, 16). A major and as yet unresolved problem is that of accurately diagnosing MASLD. In clinical practice, ultrasound is the safest, most accessible, and most cost-effective imaging test; however, its low sensitivity makes it inadequate for distinguishing between simple steatosis and steatohepatitis. Liver biopsy is the current gold standard diagnostic tool, but there is no consensus on its routine use (17), due to potential complications and the high prevalence of MASLD, which hampers large-scale implementation. Consequently, increasing attention is being paid to disease prevention, both to reduce the burden on healthcare systems and to improve clinical outcomes (8, 18).

Non-invasive methods such as serological markers and radiological techniques are currently the most widely used for predicting and detecting MASLD. Discussions continue about incorporating these methods as a first step in screening the general population for advanced liver disease, thus enabling clinicians to identify individuals who might require further, invasive investigation (19–21).

Several serological markers have been developed and validated, both for the general population and for individuals with obesity, offering a reliable assessment of the presence of steatosis, although these markers do not quantify hepatic fat content (22). Among them, the most widely used and strongly validated are the fatty liver index (FLI) and the hepatic steatosis index (HSI). Recent studies suggest that, in terms of sensitivity and specificity, the HSI more specifically detects steatosis in at-risk populations (those with diabetes and/or obesity), whereas the FLI is more effective as a screening tool for the general population (23).

MASLD has a multifactorial etiology, with significant genetic and environmental components. Effective prevention and treatment are essential to slow its progression, and lifestyle interventions such as diet and physical exercise appear to play a significant role in this respect (24, 25). Nevertheless, no evidence-based practical dietary recommendations for the prevention of MASLD have yet become widely accepted (26–29).

**Figure 1:**
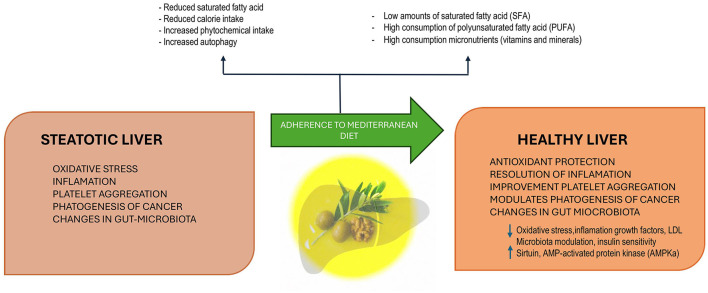
The Mediterranean diet, characterized by low saturated fatty acids (SFA), high polyunsaturated fatty acids (PUFA), and abundant micronutrients (vitamins and minerals), promotes metabolic benefits through reduced calorie intake, increased phytochemical intake, and enhanced autophagy. These mechanisms counteract oxidative stress, inflammation, platelet aggregation, and cancer pathogenesis while modulating gut microbiota. By decreasing oxidative stress, inflammation, growth factors, and LDL, and improving insulin sensitivity and microbiota composition, the Mediterranean diet enhances antioxidant protection, resolves inflammation, improves platelet function, and modulates cancer pathogenesis, contributing to liver health.

The effectiveness of dietary modifications on MASLD depends not only on calorie restriction but also on the type of calories consumed (24, 30, 31). The Mediterranean diet (MedDiet), which is characterized by a high intake of vegetables, fruits, beans, lentils, nuts, whole grains, and fish, might reduce the risk of MASLD, improve cardiometabolic health, and mitigate the adverse outcomes associated with the disease (32–34). This effect appears to be partly attributable to its high content of antioxidants and fiber, low levels of saturated fats and animal protein, and a balanced ratio of omega-3 to omega-6 fatty acids (35). The MedDiet is considered the most suitable dietary approach for patients with MASLD, primarily due to its impact on reducing oxidative stress, modulating gut microbiota and reactive oxidative species (ROS) levels, and activating autophagy (Figure 1) (35–37). Furthermore, adherence to the MedDiet is associated with improved metabolic regulation and, potentially, with the modulation of mitochondrial proteins such as Sirtuin 4, which play a role in managing oxidative stress and inflammation pathways (38). These combined effects underscore the potential of the MedDiet for the prevention and treatment of MASLD.

The few studies conducted on the MedDiet and its impact on MASLD have used widely varying designs, making it difficult to compare the information reported (39–41). Furthermore, research data for real, multi-pathological populations, including extensive long-term follow-up, are almost non-existent (41, 42).

To our knowledge, no previous large-scale, long-term population-based analysis has evaluated the effects of adherence to the MedDiet on the prevention and progression of MASLD. The aim of the present study is to investigate the long-term association between MedDiet adherence and HSI outcomes among participants in the PREvención con DIeta MEDiterránea (PREDIMED) trial.

## Materials and methods

The PREDIMED randomized clinical trial was conducted in Spain to determine the effect of a MedDiet supplemented with either extra virgin olive oil (EVOO) or nuts on the prevention of cardiovascular disease in men and women at high vascular risk, compared to a low-fat diet (43, 44). PREDIMED is registered on the Current Controlled Trials Register as ISRCTN 35739639 (http://www.predimed.es).

The study protocol complied with the principles of the Declaration of Helsinki and was approved by the Institutional Review Boards at all the recruiting centers involved. Signed informed consent was obtained from every participant.

## Study design and sample

The design, population, and methods of the PREDIMED trial have been published previously (45). In short, 7,447 men (aged 55–80 years) and women (aged 60–80 years), without documented cardiovascular disease at baseline, were recruited into the study. All presented either T2D or at least three of the following cardiovascular risk factors: current smoking habit, hypertension (blood pressure >140/90 mmHg or treatment with antihypertensives), elevated plasma low-density lipoprotein cholesterol (>160 mg/dl or treatment with lipid-lowering agents), low plasma high-density lipoprotein cholesterol (< 50 mg/dl in women and < 40 mg/dl in men), and body mass index ≥25 kg/m^2^ or family history of early coronary heart disease. All those with a history of cardiovascular disease, any serious chronic disease, or a low predicted probability of changing dietary habits (according to the stages of change model) were excluded from the trial.

The present study included 3,145 patients from the PREDIMED trial, with baseline data and 5 years of longitudinal follow-up data using yearly repeated measurements allowing to calculate the incidence and severity of HSI. At baseline, all participants reported baseline alcohol consumption of < 20 g/day (women) or < 30 g/day (men).

## Study variables

Data for the following variables were collected: age, sex, HSI, BMI, adherence to the MedDiet, fasting glucose, total cholesterol, pattern of physical activity, total calorie intake, and alcohol consumption. Data on educational background and the presence or otherwise of T2D, hypertension, hypercholesterolemia, and smoking habits were also recorded.

The HSI equation and its interpretation are shown in Annex 1.

### Physical exercise and adherence to the Mediterranean diet

Adherence to the MedDiet was assessed by the Mediterranean Diet Adherence Screener (MEDAS), a validated 14-item questionnaire (46, 47) that presents 12 questions on food consumption frequency and 2 on food consumption habits characteristic of the MedDiet in Spain. The final score that can be obtained with this questionnaire ranges from 0 to 14 points.

Physical activity (amount and intensity) was recorded using the Minnesota Leisure Time Physical Activity Questionnaire and measured in metabolic equivalents per year (48, 49). The validated Spanish version of the questionnaire includes 67 different activities and expresses the average metabolic rate as kJ/day (50, 51), based on the energy expenditure associated with these activities.

## Statistical analysis

The study variables are summarized by percentages (categorical variables) or by means and standard deviations (quantitative variables). Baseline measurements were compared using analysis of variance (ANOVA) for the quantitative variables and the chi-square test for the qualitative ones. Yearly changes in the variables during the 5-year follow-up were evaluated using repeated measures ANOVA with Greenhouse–Geisser correction for the numerical variables and the chi-square test for the qualitative ones. The longitudinal association between repeated measures of the HSI and adherence to the MedDiet was analyzed using linear mixed models with random intercept. In the models, the HSI was taken as a dependent variable. The independent variables were follow-up time and adherence to the MedDiet (both with random slopes). Adjustment for the remaining study variables was performed as follows: unadjusted (Model 1), age- and sex-adjusted (Model 2), and all study variables (including the PREDIMED intervention group)-adjusted (Model 3). All models reflected the interaction between follow-up time and MedDiet adherence, with the inclusion of a multiplicative interaction term (time x MedDiet adherence). All statistical tests were two-tailed with a significance level of 0.05 and a confidence level of 95% and were performed with Stata 18.0 software.

## Results

The mean age of the 3,145 participants was 67.2 years (± 6.2 SD); 57% were female, and 75% had at least primary education. The baseline characteristics of the intervention group and the low-fat diet recommendation group are shown in Table 1. There were no significant baseline differences between the randomized groups in terms of HSI, BMI, or the prevalence of hypertension, T2D, or hypercholesterolemia. Notably, 85% were non-smokers, and the mean BMI was 29.81 (SD 3.62), which represents overweight. The baseline prevalence of cardiometabolic disorders was T2D 47%, hypercholesterolemia 70%, and hypertension 84%.

**Table 1:** Clinical and baseline epidemiological characteristics according to the intervention and control groups of the PREDIMED trial.

**Characteristics**	**Mediterranean diet EVOO (*n =* 1124)**	**Mediterranean diet nuts (*n =* 966)**	**Low-fat diet (*n =* 1055)**
Age, years	67 (6.3)	67 (6)	68 (6.3)
Women	58	52.7	58.8
Hepatic Steatosis Index	41.1 (6.1)	52.6 (372.9)	41.7 (7.6)
Body mass index (kg/m^2^)	29.7 (3.4)	29.6 (3.5)	30.1 (3.9)
Mediterranean diet adherence	4.6 (4.6)	5 (4.5)	4.6 (4.4)
Alcohol intake (g/day)	9.3 (15.9)	10.4 (17.1)	8.5 (14.7)
Total energy intake (Kcal/day)	2328 (590)	2353 (602)	2269 (602)
Physical activity (METs-min/day)	253 (242)	272 (257)	220 (245)
Glucose (mg/dl)	121 (40)	118 (40)	121 (41)
Total cholesterol (mg/dl)	214 (38.3)	210 (36.3)	210.4 (38.5)
Type-2 diabetes mellitus	47.8	43.6	47.9
Hypercholesterolemia	71	71	69
Hypertension	82.2	85.5	84
Current smokers	14	15.1	14.6
Primary studies	73.4	74.3	78.6
Secondary studies	16.6	14.4	13.7
University studies	3.9	4.6	2.8

^*^The values are percentage or mean (standard deviation). EVOO, extra virgen olive oil; PREDIMED, PREvención con DIeta MEDiterránea; MedDiet, Mediterranean Diet.

Table 2 shows the significant changes observed in these values during the follow-up period. In short, the HSI decreased by 0.6 units (*p* < 0.001), while adherence to the MedDiet increased from 8.84 to 10.55 units (*p* < 0.001). Significant decreases were observed in alcohol intake (from 9.52 to 7.76 g/day, *p* < 0.001) and energy intake (from 2,348.37 to 2,160.04 Kcal/day, *p* < 0.001), and also in the prevalence of current smoking (from 14.53% to 5.85%, *p* < 0.001), hypercholesterolemia (from 70.37% to 17.48%, *p* < 0.001), and hypertension (from 83.82% to 23.56%, *p* < 0.001). The average glucose level decreased during the first 2 years, but increased in the third, fourth, and 5th years.

**Table 2:** Description of characteristics throughout the follow-up.

**Characteristics**	**Baseline**	**Year 1**	**Year 2**	**Year 3**	**Year 4**	**Year 5**	***p***

	***n** =* **3145**	***n** =* **2497**	***n** =* **1813**	***n** =* **1982**	***n** =* **1205**	***n** =* **1395**	
Hepatic steatosis index	40.85 (40.73–40.97)	40.64 (40.51–40.78)	40.66 (40.50–40.82)	40.73 (40.58–40.88)	40.58 (40.38–40.78)	40.25 (40.06–40.43)	< 0.001^†^
Mediterranean diet adherence (0–14)	8.84 (8.79–8.91)	10.32 (10.26–10.37)	10.46 (10.40–10.52)	10.44 (10.38–10.50)	10.52 (10.45–10.58)	10.55 (10.48–10.62)	< 0.001^†^
Alcohol intake (g/day)	9.52 (9.26–9.78)	8.68 (8.40–8.96)	8.67 (8.37–8.96)	8.17 (7.87–8.47)	7.96 (7.63–8.29)	7.76 (7.41–8.11)	< 0.001^†^
Total energy intake (Kcal/day)	2348.37 (2332.74−2364.00)	2324.01 (2307.28–2340.74)	2299.62 (2281.75–2317.49)	2266.64 (2248.80–2284.47)	2213.67 (2193.92–2233.43)	2160.04 (2139.36–2180.72)	< 0.001^†^
Physical activity (METs-min/day)	253.65 (246.64–260.67)	249.9 (242.41–257.40)	248.14 (240.13–256.16)	269.66 (261.66–277.65)	268.55 (259.68–277.41)	251.65 (242.39–260.91)	< 0.001^†^
Glucose (mg/dl)	119.26 (118.40–120.11)	118.55 (117.66–119.44)	117.80 (116.78–118.81)	120.21 (119.25–121.17)	120.35 (119.19–121.50)	121.85 (120.71–122.98)	< 0.001^†^
Total cholesterol (mg/dL)	212.31 (211.43–213.19)	208.31 (207.40–209.23)	206.27 (205.22–207.32)	203.91 (202.92–204.90)	202.32 (201.12–203.52)	199.40 (198.23–200.58)	< 0.001^†^
Hypercholesterolemia (%)	70.37	29.67	20.24	19.13	17.76	17.48	< 0.001^‡^
Hypertension (%)	83.82	38.35	31.94	23.90	23.25	23.56	< 0.001^‡^
Current smokers (%)	14.53	11.00	8.61	7.59	5.78	5.85	< 0.001^‡^

^†^Repeated measures ANOVA with Greenhouse-Geisser correction.

^‡^Chi-square test.

Table 3 shows the relationship between the study variables and the HSI. The interaction (product*term) assessing the effect of the randomized intervention on changes over time in HSI was not statistically significant in any of the three models, yielding *p*-values of 0.087, 0.097, and 0.230 in Models 1, 2, and 3, respectively. In all cases, however, the HSI decreased over time. As additional variables were incorporated into the models (from Models 1–3), the time coefficient increased slightly but remained negative. Thus, the association is consistent, even after adjusting for age, sex, alcohol intake, physical activity, and other covariates. Although the effect of time was attenuated in Model 3, it remained statistically significant. Additionally, significant inverse associations were observed between the HSI and the level of adherence to the MedDiet.

**Table 3:** Influence of study variables on hepatic steatosis index.

**Variables**	**Model 1**		**Model 2**		**Model 3**	

	**coefficient (**β**)**	**95% CI**	**coefficient (**β**)**	**95% CI**	**coefficient (**β**)**	**95% CI**
Time since baseline (years)	**−0.242**	**−0.435**, **−0.049**	**−0.240**	**−0.433**, **−0.047**	**−0.210**	**−0.414**, **−0.007**
MedDiet adherence (0–14)	**−0.097**	**−0.148**, **−0.046**	**−0.093**	**−0.144**, **−0.042**	**−0.075**	**−0.128**, **−0.021**
Time x MedDiet adherence	0.016	−0.002, 0.035	0.016	−0.003, 0.034	0.012	−0.007, 0.031
Age at baseline (years)			**−0.166**	**−0.198**, **−0.134**	**−0.172**	**−0.202**, **−0.141**
Alcohol intake (g/day)					−0.001	−0.009, 0.007
Total energy intake (per 100 Kcal/day)					**0.025**	**0.009, 0.041**
Physical activity (per 100 METs-min/day)					**−0.096**	**−0.128**, **−0.064**
Current smoker					−0.305	−0.676, 0.066
Glucose (mg/dl)					**0.020**	**0.017, 0.022**
Hypercholesterolemia					−0.160	−0.349, 0.030
Hypertension					0.162	−0.023, 0.347
**PREDIMED trial arm:**						
Mediterranean diet + EVOO					**−0.620**	**−1.077**, **−0.164**
Mediterranean diet + nuts					**−0.881**	**−1.366**, **−0.406**

Model 1: Random effect linear regression with a random inteRcept and a random slope for time since baseline and Mediterranean diet adherence.

Model 2: Model 1 further adjusted by sex and age at baseline (continuous).

Model 3: Model 2 further adjusted by alcohol intake (continuous), total energy intake (continuous), physical activity (continuous), glucose (continuous), smoking (binary), hypercholesterolemia, hypertension and PREDIMED intervention group: Low fat intake (reference).

Mediterranean diet + extra virgin olive oil (EVOO) and Mediterranean diet + mixed nuts.

Statistically significant results are shown in bold (p < 0.05).

HSI values were significantly lower among the participants who entered the PREDIMED trial at an older age (Model 2: β = −0.166; 95% CI: −0.198, −0.134; Model 3: β = −0.172; 95% CI: −0.202, −0.14) and those who engaged in greater daily physical activity (β = −0.001; 95% CI: −0.0013, 0.0006). By contrast, values were significantly higher in participants with higher blood glucose levels (β = 0.020; 95% CI: 0.017, 0.022) and in those with a higher daily calorie intake (β = 0.0003; 95% CI: 0.0001, 0.0004).

In Model 3, the MedDiets supplemented with either EVOO or nuts were both inversely associated with the HSI, with statistical significance. Among the participants who consumed the MedDiet with EVOO, the HSI was 0.620 units (β = −0.620, 95%CI: −1.077, −0.164) less than in the reference group (low-fat diet), while for those who consumed the MedDiet with nuts, the corresponding difference was 0.881 units (β = −0.881, 95% CI: −1.366, −0.406).

## Discussion

This study investigates the relationship between adherence to the MedDiet and the presence and progression of MASLD, as assessed by longitudinal changes in the HSI, in a large cohort of patients with cardiometabolic disorders followed for 5 years. Better adherence to the MedDiet is associated with a lower HSI. Specifically, the linear regression model, adjusted for potential confounders, shows that each one-point increase in the MEDAS score is associated with a 0.07-unit reduction in the HSI.

These findings are consistent with previous research in this field, suggesting that adherence to the MedDiet may positively affect cardiometabolic health and liver function (52–55). For instance, Baratta et al. demonstrated that adherence to the MedDiet was associated with a lower prevalence of liver steatosis (low adherence vs. high adherence: 96.5% vs. 71.4%; *P* < 0.001) (56). Additionally, Ryan et al. conducted a 6-week crossover dietary intervention study with the MedDiet and observed a significant reduction in liver steatosis and improved insulin sensitivity in insulin-resistant patients with MASLD (40).

In another study, Abenavoli et al. found that adherence to the MedDiet resulted in a sustained reduction in hepatic steatosis rates for up to 1 year (57). Similarly, a 6-month single-arm counseling intervention with MedDiet decreased the percentage of patients with steatosis grade 2 or higher from 93% to 48% (58). Moreover, our findings align with the results of the cross-sectional analysis of two studies conducted on over 13,000 participants across two population-based cohorts in Switzerland and England. The results showed that higher adherence to the MedDiet was associated with a lower prevalence of hepatic steatosis, assessed using abdominal ultrasound and FLI in the Fenland study (England), and the FLI and the MASLD score in the CoLaus study (Switzerland) (59). Furthermore, our findings are also consistent with two analyses conducted within the PREDIMED and PREDIMED-Plus cohorts. In the PREDIMED trial, participants with demographic and clinical profiles similar to those in the present study showed a direct correlation between liver damage (defined as >50th percentile FLI) and both a pro-inflammatory dietary pattern and lower adherence to the MedDiet in obese individuals (60). In the PREDIMED plus study, which included 278 participants from the Navarra-Nutrition node, adherence to the MedDiet negatively correlated with the HSI score, in both men (*r* = −0.18, *p* = 0.032) and women (*r* = −0.19, *p* = 0.027) (61). In a study of 276 PREDIMED-Málaga participants, the rate of change in FLI among the control group increased over time (1.13 ± 0.4; *p* = 0.006), suggesting that the MedDiet intervention delayed or slowed the natural progression of MASLD (62). A recent meta-analysis of six randomized controlled trials (total sample size: 250 participants) reported that the Mediterranean diet significantly reduced FLI compared to the control diet (standardized mean difference: −1.06, 95% CI: −1.95 to −0.17; *p* = 0.02) (63). In our study, the average HSI among participants who adhered to the MedDiet significantly decreased over the follow-up period, with a difference of −0.6 units between the first and fifth years.

The MedDiet is known to have beneficial health effects, especially in helping prevent cardiovascular disease (64–67). These benefits are probably mediated through the reduction of systemic inflammation, independent of changes in lipid levels or body weight (68). This association underscores the anti-inflammatory properties of the Mediterranean dietary pattern as a critical mechanism in mitigating cardiovascular risk and disease progression. These mechanisms are likely to exert also an impact on the development, prevention, and treatment of MASLD (43, 69–71). A recent publication highlighted the important role of diet in the preventive treatment of MASLD, although it also pointed out that no large-scale long-term population studies have yet been conducted to test the effects of structured lifestyle intervention programs on preventing this disease (72). The favorable outcomes achieved by the MedDiet for MASLD in our study may be explained, at least in part, by the fact that this dietary pattern is rich in polyphenols, carotenoids, vitamins, and other biomolecules that have anti-inflammatory and antioxidant effects (73–75). This characteristic seems to be relevant, since inflammation and oxidative stress are involved in the pathogenesis of MASLD (76). Moreover, the monounsaturated fatty acids present in the MedDiet, in olive oil, for example, improve the lipid profile, which is consistent with the significant decrease in the HSI observed in the intervention groups (MedDiet plus EVOO or nuts).

As well as the direct relation observed between the HSI and adherence to the MedDiet, it is also indirectly related to the participants' age of entry into the study and their level of physical activity. Thus, for every additional point scored for physical activity, the HSI decreased by 0.096 units, a statistically significant relation. This association aligns with previous research findings, according to which physical activity is helpful for patients with MASLD because it improves their metabolic condition (77). However, for this improvement to be effective, physical activity must be accompanied by an appropriate dietary intervention, such as the MedDiet, including antioxidants such as EVOO (78). With respect to the participants' age at entry, for each additional year at baseline, the HSI improved (i.e., fell) by 0.2 units. Although age is a predisposing factor for MASLD, we believe the latter finding may also reflect the baseline characteristics of our participants, in that those who met the inclusion criteria and were younger probably presented a higher level of deterioration than those who started later and would have been taking better care of themselves. If this were so, it would not be surprising that participants who began the study at an older age had a better prognosis and a lower HSI.

Our results show that the HSI is positively associated with the baseline glucose level and kilocalorie intake. Specifically, for every additional unit of the blood glucose level, the HSI increases (significantly) by 0.02 units. This could be because the accumulation of intrahepatic fat is associated with that of intrahepatic ceramides and diacylglycerols, which inhibit insulin signaling and, therefore, promote hepatic insulin resistance (79). With this increased hepatic insulin resistance, MASLD negatively affects glycaemic control in patients with T2D (78). In fact, patients with both T2D and MASLD generally require more intensive antidiabetic therapies to achieve optimal glycaemic control compared to patients with T2D alone (80). Regarding the relationship between the HSI and calorie intake, for every additional calorie consumed, the HSI increased by 0.025 units. Nevertheless, the participants' calorie intake decreased significantly during the 5-year follow-up. As is well known, excessive calorie consumption promotes obesity and related comorbidities such as MASLD, a relation that is corroborated in our study results and those of other investigations (81–84).

In our study, all participants reported baseline alcohol consumption within the defined limits of < 20 g/day for women and < 30 g/day for men. These thresholds align with widely accepted guidelines for low-to-moderate alcohol intake in clinical research. However, recent evidence suggests that even very moderate alcohol consumption, such as 66–96 g/week, may increase the risk of significant fibrosis progression compared to no or low alcohol consumption. This may be explained by the role of alcohol metabolism in exacerbating oxidative stress and inflammation, which are key mechanisms driving the progression of NAFLD and MASLD (85). Among smokers, there is a strong and dose-dependent correlation between MASLD and a smoking history of >10 pack-years. Moreover, ex-smokers who quit < 10 years ago are more susceptible to the disease than those who have never smoked (86). Stratifying participants by finer consumption thresholds and by the duration of smoking cessation could provide greater clarity on the dose- and time-dependent effects of alcohol and tobacco on liver histology and fibrosis progression. These findings highlight the need for cautious interpretation of the impact of moderate alcohol consumption and the time elapsed since smoking cessation on MASLD management.

Finally, as observed above, very few observational or field studies have assessed the impact of the MedDiet on the progression of hepatic steatosis, and those available differ significantly in terms of design, sample characteristics, and means of assessing adherence to the MedDiet, which makes any comparison difficult (87–89). In general, however, these papers report a beneficial effect of the MedDiet on hepatic steatosis, which is in line with our findings. The present study, moreover, was carried out on a sample of over 3,000 patients aged over 67 years, and presenting high cardiovascular risk (83% were hypertensive, 70% hypercholesterolaemic, and 47% diabetic), with a mean baseline BMI >29 kg/m^2^ and an HSI >30. This population, therefore, presented challenges for the effective management of MASLD. For these patients, the MedDiet may represent a valuable therapeutic option, as it is relatively easy to sustain, in contrast to the difficulties often experienced with more restrictive interventions in achieving sustained weight loss (90, 91).

## Strengths and limitations

A multicenter randomized controlled trial study design is considered the gold standard for evaluating the efficacy and safety of dietary interventions. However, in the present case, despite the large sample size considered and the 5-year follow-up period, the study design has limitations, such as its use of an indirect estimator to measure MASLD. However, the HSI is a validated marker of hepatic steatosis that is based on a non-invasive and easy-to-determine measurement, which makes it suitable for large epidemiological studies. Previous studies have shown their results to be consistent (92–97). Additionally, the gold standard for assessing MASLD—liver biopsy—is an invasive method that cannot be implemented in large-scale epidemiological studies with predominantly healthy participants. Moreover, liver biopsies are costly and may introduce sampling errors and procedural complications (88). Another study limitation is the fact that the study sample was composed of older adults, all at high cardiovascular risk and living in the Mediterranean region. This profile limits the generalizability of our results to other age groups or ethnicities. However, as the intervention generated beneficial results in such a complex population, we would expect the results for the general population to be better still.

## Conclusion

The findings of this study suggest that adherence to the Mediterranean diet is significantly associated with a long-term reduction in hepatic steatosis in patients with multimorbidity and at high cardiovascular risk, and highlight its potential as an effective strategy for reducing liver fat accumulation, particularly in individuals at risk of MASLD. Promoting the inclusion of the MedDiet as a supplementary strategy for preventing MASLD could generate substantial health improvements in at-risk populations, including individuals with obesity, T2D, dyslipidaemia, or hypertension, and particularly among older adults.

## Data Availability

The original contributions presented in the study are included in the article/supplementary material, further inquiries can be directed to the corresponding author.

## ANNEX

**Annex 1**. Hepatic Steatosis Index (HSI). The hepatic steatosis index is based on four variables: transaminases (ALT and AST), body mass index (BMI), sex, and type 2 diabetes (T2D): *HSI = 8* × *(ALT/AST ratio) + BMI*. (+2, if female; +2, with T2D). The interpretation is as follows:

- HSI < 30, no steatosis.
- HSI > 36, hepatic steatosis.
